# Pyridostigmine improves hand grip strength in patients with myalgic encephalomyelitis/chronic fatigue syndrome

**DOI:** 10.3389/fnins.2025.1637838

**Published:** 2025-09-03

**Authors:** Ella Schlömer, Elisa Stein, Claudia Kedor, Rebekka Rust, Anna Brock, Kirsten Wittke, Carmen Scheibenbogen, Laura Kim

**Affiliations:** ^1^Institute of Medical Immunology, Charité-Universitätsmedizin Berlin, Corporate Member of Freie Universität Berlin and Humboldt-Universität zu Berlin, Augustenburger Platz, Berlin, Germany; ^2^Experimental and Research Center (ECRC), Charité-Universitätsmedizin Berlin, Corporate Member of Freie Universität Berlin and Humboldt-Universität zu Berlin, Charitéplatz, Berlin, Germany

**Keywords:** myalgic encephalomyelitis/chronic fatigue syndrome, post-COVID syndrome, hand grip strength, orthostatic intolerance, myasthenia gravis, COVID-19, pyridostigmine

## Abstract

**Background:**

Myalgic encephalomyelitis/chronic fatigue syndrome (ME/CFS) is a multisystemic disease characterized by exertional intolerance and fatigue which is often accompanied by muscle weakness and fatiguability. A study showed efficacy of the acetylcholinesterase inhibitor pyridostigmine on cardiac output in ME/CFS patients. Pyridostigmine is currently used off-label in ME/CFS and postural orthostatic tachycardia syndrome.

**Methods:**

We evaluated the effect of pyridostigmine on hand grip strength in 20 patients with post-infectious ME/CFS. Hand grip strength testing was performed ten times using an electric dynamometer and was repeated after 1 h. In a second test, 30 mg of pyridostigmine was given immediately after the first measurement. Orthostatic function was assessed using a passive standing test. Neurological examination and autoantibody testing were performed to rule out a diagnosis of myasthenia gravis.

**Results:**

All patients had reduced maximum hand grip strength with a median of 16.45 kg (IQR: 11.45 kg–22.8 kg). Hand grip strength was diminished by a median of 4.65 kg after 1 h. In contrast, 1 h after pyridostigmine administration, patients showed an improvement in maximum hand grip strength with a median increase of 2.6 kg. The maximum hand grip strength after exertion was about 1.5-fold higher with then without pyridostigmine (*p =* 0.01). The increase in heart rate from lying to standing was median 17 beats per minute without pyridostigmine (IQR: 13 beats per minute – 23 beats per minute) and 13 beats per minute (IQR: 9 beats per minute – 20 beats per minute) (*p* = 0.017) with pyridostigmine. None of the patients tested positive for myasthenia gravis specific autoantibodies.

**Conclusion:**

Pyridostigmine exerts an immediate effect on muscle strength and orthostatic function. This may be attributed to increased acetylcholine availability at neuromuscular junctions, and its augmentation of parasympathetic tone.

## 1 Introduction

Myalgic encephalomyelitis/chronic fatigue syndrome (ME/CFS) is a complex disease affecting the immune system (Sotzny et al., 2018; Maksoud et al., 2023) and the autonomic nervous system (Spence et al., 2004; Hartwig et al., 2020; Słomko et al., 2020) including the vascular regulation (van Campen et al., 2020; Newton et al., 2012). There is evidence that autoantibodies to adrenergic and acetylcholine receptors play a potential role (Spence et al., 2004; Hartwig et al., 2020; Słomko et al., 2020). The World Health Organization (WHO) categorizes ME/CFS as a neurological disorder. In the majority of cases, ME/CFS is triggered by an infection. ME/CFS has been shown in a subset of patients with post COVID syndrome (PCS), and COVID has now become one of the most frequent triggers of the disease (Kedor et al., 2022). Hallmark symptoms of ME/CFS include severe physical and cognitive fatigue unrelieved by rest along with other symptoms including sleeping disorders, muscle pain, and disturbed neurocognitive function (Nacul et al., 2021). Post-exertional malaise (PEM) is characteristic for the condition, describing a worsening of the symptoms after physical or cognitive everyday activities (Cotler et al., 2018).

The diagnosis of ME/CFS currently relies on clinical assessments using established criteria most often the Canadian Consensus Criteria (CCC) or the less strict Institute of Medicine (IOM) criteria (Carruthers et al., 2003; Committee on the Diagnostic Criteria for Myalgic Encephalomyelitis/Chronic Fatigue Syndrome et al., 2015). Exercise testing in patients with PCS and PEM as well as ME/CFS provides evidence of a diminished exercise capacity compared to healthy controls (Joseph et al., 2021). Reduced muscle strength, particularly in hand grip and quadriceps strength measurements, has been consistently demonstrated in this population (Jäkel et al., 2021; Nacul et al., 2018). Hand grip strength (HGS) measurement has emerged as a useful complimentary, objective tool to assess muscle fatigue and fatiguability in ME/CFS patients (Jäkel et al., 2021; Legler et al., 2023). Notably, diminished hand grip strength correlates with prognosis and key ME/CFS symptoms, underscoring the significance of skeletal muscle pathophysiology (Legler et al., 2023; Paffrath et al., 2024).

While the precise mechanisms underlying the muscular dysfunction remain incompletely understood, biopsies from muscles revealed changes in muscle morphology and mitochondrial function, including decreased oxidative phosphorylation capacity, and lower mitochondrial content (Scheibenbogen and Wirth, 2025) While functional impairment could be observed in both PCS and ME/CFS patients, ME/CFS patients exhibited additional morphological changes in mitochondria cristae and size (Bizjak et al., 2024; Appelman et al., 2024). Additionally, there was a shift toward more fatigable glycolytic muscle fibers and a metabolic shift away from oxidative metabolism, along with lower levels of muscle creatine and S-adenosylmethionine. Exercise induced muscle tissue damage with increased atrophy, focal necrosis, and immune cell infiltration (Appelman et al., 2024). Endothelial dysfunction is a recognized feature of ME/CFS and Bertinat et al. (2022) have demonstrated impaired nitric oxide (NO) production in ME/CFS patients. This could lead to vasomotor dysregulation, contributing to muscle hypoperfusion in ME/CFS. Increased muscle sodium content and changes in the microvasculature further support the concept of hypoperfusion upon exertion as underlying mechanism (Bizjak et al., 2024; Petter et al., 2022; Wirth and Scheibenbogen, 2021).

In the differential diagnosis of severe muscle fatigue, ME/CFS needs to be distinguished from myasthenia gravis (MG), a rare autoimmune disease characterized by rapid muscle weakness upon exertion due to autoantibodies targeting postsynaptic acetylcholine receptors (Kaminski and Kusner, 2018). Several studies showed that COVID-19 can trigger autoimmune diseases including MG (Restivo et al., 2020; Shah et al., 2022; Taheri et al., 2022). The Pyridostigmine (PS)-test is commonly used as diagnostic test for MG (Mantegazza and Cavalcante, 2019). PS inhibits the dismantling of acetylcholine in the synaptic cleft which leads to a higher transmitter-availability resulting in an improvement of muscle strength in MG patients (Farmakidis et al., 2018). Glikson et al. demonstrated that PS has no significant neuromuscular effects in healthy controls (Glikson et al., 1991).

Due to its potential benefits in cardiovascular regulation, PS is being prescribed off-label to ME/CFS patients, particularly those experiencing orthostatic intolerance. A first randomized controlled trial (RCT) investigating the effect of a single dose of 60 mg PS in ME/CFS has shown improvements in peak exercise oxygen uptake after 1 h, which were attributed to increased cardiac output and enhanced right ventricular filling pressures (Joseph et al., 2022). Furthermore, PS has been shown to reduce heart rate and alleviate symptoms in patients diagnosed with postural orthostatic tachycardia syndrome (POTS) (Raj et al., 2005). Orthostatic intolerance is another key symptom of cardiovascular dysregulation in ME/CFS with up to 25% of the patients suffering from comorbid POTS (van Campen et al., 2018).

The aim of this study was to evaluate the immediate effect of PS on HGS and orthostatic tolerance in patients with ME/CFS.

## 2 Materials and methods

### 2.1 Patients

This study was conducted at the outpatient clinic of the Institute of Medical Immunology, Charité – Universitätsmedizin Berlin. Thirty-nine ME/CFS patients were screened between November 2023 and August 2024. Twenty of them fulfilled the inclusion criteria for this study.

Patients between 18 and 65 years were included in this study. The diagnosis of ME/CFS was based on the CCC (Carruthers et al., 2003). Muscle fatigue was defined by an impaired HGS according to cut-off values from the hand dynamometer manufacturer as shown in Table S1. Exclusion criteria included current pregnancy, a Bell Score below 30, inability to speak German, and significant comorbidities that preclude the ME/CFS diagnosis such as cancer or multiple sclerosis (MS) (Bateman et al., 2021).

All participants provided written informed consent prior to inclusion. The study received approval from the Ethics Committee of Charité—Universitätsmedizin Berlin, adhering to the 1964 Declaration of Helsinki and its subsequent amendments (protocol EA2/170/23, date of approval: 29 September 2023).

### 2.2 Procedures

#### 2.2.1 Study design

This study utilized a within-subject design to assess the effects of PS on HGS and orthostatic function in patients. During the baseline visit, HGS and orthostatic function were assessed without PS administration to establish a control measure. At the second visit, where participants received a single dose of 30 mg PS, the same tests were performed both before and 1 h after PS administration. The test was not conducted under non-fasting conditions. Patients were closely observed during and following the administration of PS, and no immediate adverse effects occurred.

#### 2.2.2 HGS measurement

HGS of the dominant hand was measured using a digital hand dynamometer (EH101, Deyard, Shenzhen, China). Prior to the measurements, patients was shown the proper use of the dynamometer. For the HGS measurement, patients were seated upright facing a standard table. The forearm of the dominant hand was placed on the table in full supination while holding the dynamometer. Under supervision and verbal encouragement, patients exerted maximum force on the dynamometer handle for 3 s, followed by a 5 s relaxation period. The dynamometer displayed the highest value achieved within the 3 s exertion period (measured in kilograms) and this single value was recorded. This was repeated ten times. The highest value out of the ten repetitions was documented as maximum HGS (Fmax). The mean HGS (Fmean) was calculated for each session (Jäkel et al., 2021). Normal ranges of HGS are defined by age- and sex-adjusted values according to the digital hand dynamometer’s manufacturer, as shown in the Supplementary material. The HGS testing was repeated after 60 min.

#### 2.2.3 Passive standing test

An automated blood pressure cuff (Philips EarlyVue VS30) was placed on the patient’s right upper arm to record blood pressure (BP) and HR. Resting measurements were taken with the patients in a supine position. Subsequently, patients were asked to stand straight with their shoulders leaned against a wall and their heels one step away from the wall. Patients were asked to hold this position for 10 min. BP and HR were recorded every minute for 10 min. The test was terminated earlier if the patient was unable to continue standing due to severe orthostatic symptoms. Throughout the test, patients were continuously monitored and any symptoms were recorded. Patients were diagnosed with POTS if they exhibited a sustained pulse increase of more than 30 bpm (or exceeding 120 bpm) within 10 min of standing, accompanied by clinical signs of orthostatic intolerance persisting for at least 3 months, in the absence of orthostatic hypotension (Vernino et al., 2021). POTS is defined by orthostatic symptoms such as dizziness, palpitations, and headaches lasting for a minimum of 3 months accompanied by an abnormal increase in the standing from supine heart rate (HR) (Sheldon et al., 2015).

#### 2.2.4 Patient-reported outcomes

Patients’ health status and related quality of life was assessed using the 36-Item Short-Form Survey (SF-36), with scores ranging from 0 to 100, with 100 indicating no limitations (Ware, 2000). Additionally, disease-related disability was scored according to the Bell score, rating the restriction in daily functioning on a scale from 0 to 100, with 100 indicating no restriction (Bell, 1995). Both questionnaires were assessed at both visits.

#### 2.2.5 Besinger score

Muscle strength was further assessed using the Besinger score, a tool commonly used to monitor the progression of MG. This score evaluates specific muscle groups, with grades ranging from 0 (normal) to 3 (severe), where higher scores indicate greater muscle weakness. The tested muscle groups included ocular, bulbar, limb, and axial muscles, each assessed individually. During the evaluation, patients were instructed to perform specific actions, such as holding an arm outstretched or lifting the head, and to sustain the position for a pre-defined duration. The examiner recorded the time the position was maintained before fatigue occurred, assigning a grade based on the observed muscle weakness. The total Besinger score was calculated by summing the scores for all tested muscle groups and dividing by the number of criteria assessed, providing an overall measure of muscle strength (Berlit, 2006).

#### 2.2.6 MG autoantibodies

Autoantibodies against the muscle nicotinic acetylcholine receptor (MN-AChR) were determined using enzyme immunoassay, muscle-specific receptor tyrosine kinase (MuSK)-antibodies with enzyme-linked immunosorbent assay, transmembrane protein Lrp4-antibodies with immunofluorescence assay, and voltage-gated calcium channels (VGCC)-autoantibodies with radioimmunoassay.

### 2.3 Statistical analysis

Statistical analyses were conducted using R version 4.3.0 and RStudio version 2023.03.1, with data visualization performed using the ggplot2 package (version 3.5.0). The Wilcoxon signed-rank test was applied to compare paired measurements across time points, as the data were not normally distributed. Since this non-parametric test compares paired values within the same subject, interindividual differences such as age and sex are inherently controlled for. Spearman’s rank correlation coefficient was then used to assess associations between variables. A *p*-value of <0.05 was considered statistically significant.

## 3 Results

### 3.1 Patients characteristics

Seven male and 13 female patients with post-infectious ME/CFS were included in this study, with a median disease duration of 25 months (IQR: 16.5–28.5 months) at the time of study inclusion. Two patients had respiratory tract infections as the triggering event, while all the others had developed ME/CFS following SARS-CoV-2 infection.

Patients exhibited marked impairment in physical function, with a median SF-36 Physical Functioning score of 30 (IQR: 20–45), and substantial fatigue, reflected by a median SF-36 Vitality score of 20 (IQR: 10–40). The median SF-36 Pain score was 41 (IQR: 22–41), indicating a high pain burden. Overall, physical limitations were further emphasized by a median Bell score of 40 (IQR: 30–40), corresponding to moderate to severe functional disability. Detailed patient characteristics are presented in Table 1.

**Table 1:** Patient characteristics at baseline.

Characteristics	Median (IQR)
	pre PS
Sex (*n*, %)	Female 13/20 (65%) Male 7/20 (35%)
Trigger infection (*n,* %)	COVID-19 18/20 (90%) Upper respiratory tract infection 2/20 (10%)
Age (years)	42.5 (35.5–50.0)
BMI	23.85 (22.75–29.78)
Disease duration (months)	25.0 (16.5–28.5)
Besinger score	
Arm outstretched time (points)	1.0 (1.0–1.0)
Leg outstretched time (points)	2.0 (2.0–2.5)
Head holding time (points)	1.5 (1.0–2.0)
Ptosis (points)	0.0 (0.0–0.0)
Diplopia (points)	0.5 (0.0–1.0)
Total score (points)	0.9 (0.7–1.3)
SF36	
Physical function	30.0 (20.0–45.0)
Role limitations physical	0.0 (0.0–0.0)
Role limitations emotional	67.0 (0.0–100.0)
Vitality	20.0 (10.0–40.0)
Mental health	72.0 (52.0–80.0)
Social function	25.0 (13.0–25.0)
Pain	41.0 (22.0–41.0)
General health	25.0 (15.0–40.0)
Bell-score	40.0 (30.0–40.0)
Medication (*n*)	Fexofenadine (*n* = 3), low-dose naltrexone (*n* = 3), levothyroxine (*n* = 3), pregabalin (*n* = 2), candesartan (*n* = 2), metformin (*n* = 2), N-acetylcysteine (*n* = 1), apixaban (Eliquis) (*n* = 1), venlafaxine (*n* = 1), semaglutide (Ozempic) (*n* = 1), tamsulosin (*n* = 1), rosuvastatin (*n* = 1), escitalopram (*n* = 1), pantoprazole (*n* = 1), amitriptyline (*n* = 1), acetylsalicylic acid (*n* = 1), metoprolol (*n* = 1), and bupropion (*n* = 1).

BMI, body mass index; SF36, 36-Item Short Form Survey; 1 h post PS, 1 h after treatment with 30 mg PS.

### 3.2 HGS measurement

**Figure 1:**
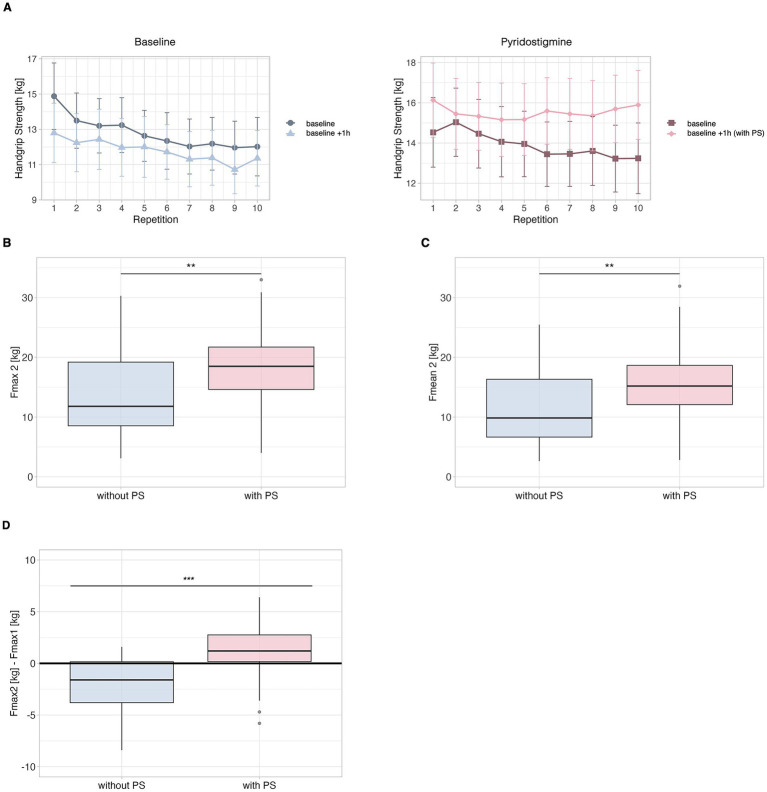
Hand grip strength (HGS) in patients with post-infectious ME/CFS (*n* = 20). Handgrip strength (HGS) was assessed in two separate sets, each consisting of ten consecutive measurements, with a 1 h rest period between sets. Mean and maximum HGS were calculated for each measurement set. The visit without pyridostigmine (PS) is shown in blue, while the visit with 30 mg of PS administered after the first measurement set is shown in pink. **(A)** Course of the single HGS values, left: without PS, right: with PS. **(B)** Maximum force (Fmax2) of the second HGS measurement set, with and without PS. **(C)** Mean force (Fmean2) of the second HGS measurement set, with and without PS. **(D)** Difference in maximum force (Fmax) between the first and second measurement set. Group comparisons were performed using the Wilcoxon matched paired-signed rank test with significance levels indicated as * *p* < 0.05, ** *p* < 0.01, *** *p* < 0.001.

As depicted in Figure 1A, HGS exhibited a linear decrease over the course of the ten measurements without PS but remained stable following PS administration. Further patients had a lower Fmax and Fmean HGS in the second session compared to the first without PS, while it increased after PS. In total, 16 of 20 patients had an improved HGS after 1 h. In four of 20 patients Fmax or Fmean did not increase following PS administration.

At the first visit, patients presented with a median Fmax of 16.5 kg (IQR: 11.5 kg–22.8 kg). After 60 min Fmax2 was significantly lower with a median of 11.8 kg (IQR: 8.2 kg–19.3 kg) (*p* = 0.007), which corresponds to a reduction of 32%. Similarly, Fmean showed a significant decline after 1 h, dropping from a median of 11.7 kg (IQR: 7.8 kg–19.3 kg) to 9.9 kg (IQR: 6.3 kg–16.4 kg = 16% decrease) (*p* = 0.012) indicating impaired strength recovery.

During the second visit, 30 mg of PS were given immediately after the first ten measurements. In contrast to the initial measurement, Fmax then increased from a median of 15.9 kg (IQR: 11.4 kg–22.9 kg) to 18.5 kg (IQR: 14.2 kg–21.9 kg) 1 h after PS administration as demonstrated in Figure 1A. Similarly, PS administration resulted in a significant improvement in Fmean2, with an increase of 1.9 kg (12.6%) (*p* = 0.03), increasing from a median of 13.3 kg (IQR: 8.3 kg–19.1 kg) to 15.20 kg (IQR: 11.3 kg–18.9 kg). Thus, the HGS after 1 h (Fmax2) was about 1.5-fold higher with versus without PS (*p* = 0.01) as demonstrated in Figure 1B. Similarly, the Fmean2 was also about 1.5-fold higher with versus without PS (*p* = 0.002) as demonstrated in Figure 1C.

Figure 1D illustrates the Fmax2 decline in the absence of PS and the Fmax2 increase after PS (*p =* <0.001).

### 3.3 Passive standing test (PST)

**Figure 2:**
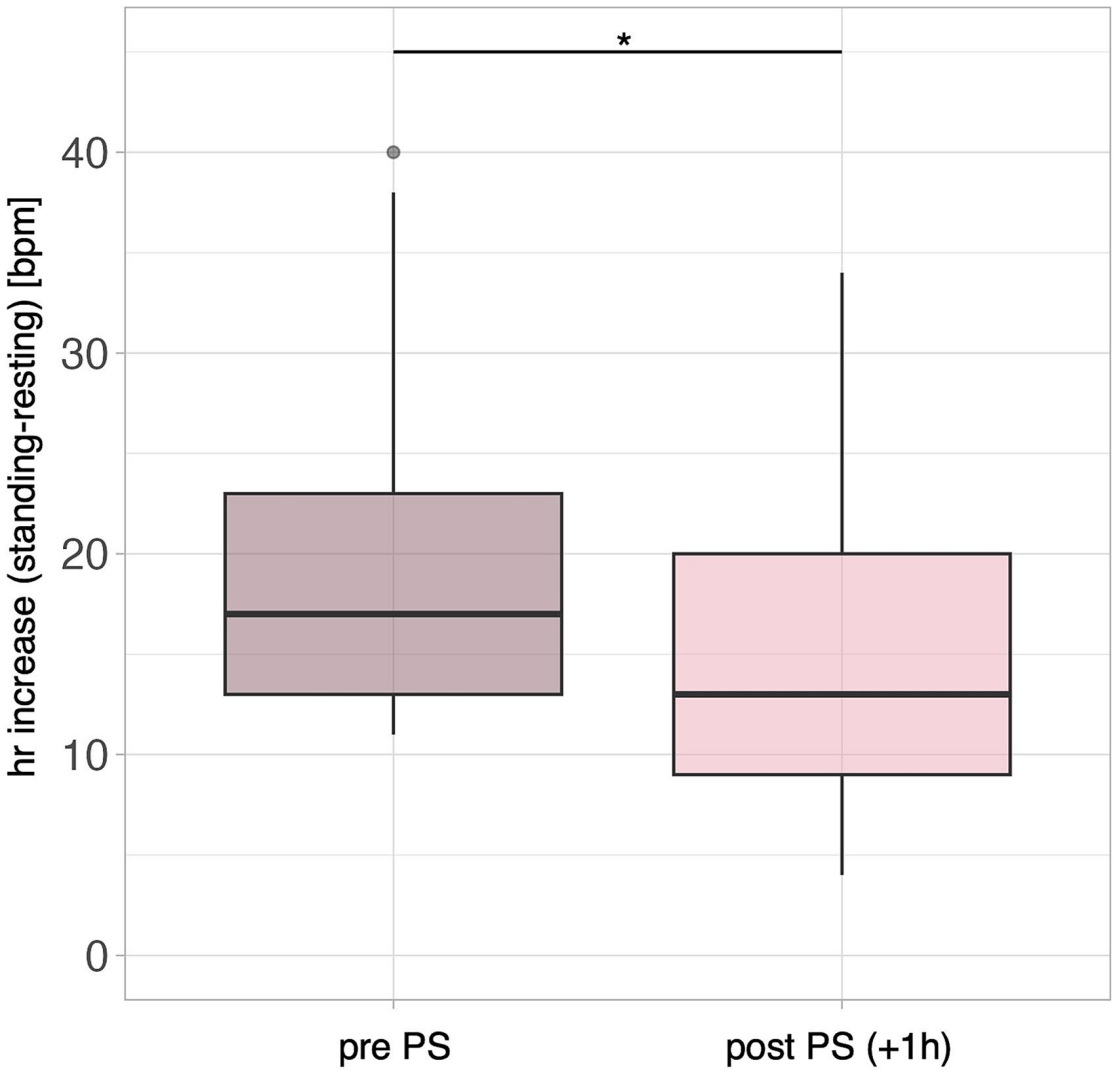
Median HR-increase when changing from a supine to a standing position during the PST in post-infectious ME/CFS patients (*n* = 13). Left: HR-increase pre-PS-administration; right: HR-increase 1 h post PS-administration. Dark blue indicates the measurement after PS-administration. Group comparisons were performed using the Wilcoxon matched paired-signed rank test with significance levels indicated as * *p* < 0.05.

The PST to assess orthostatic function was performed in 13 out of 20 patients. Seven patients declined the PST due to concerns about symptom exacerbation and because they already had a previous PST. Among them, two fulfilled diagnostic criteria for POTS with a persistent orthostatic heart rate increase of 34 bpm and 30 bpm, respectively, and orthostatic symptoms. No patient had a maximal HR over 120 bpm. Prior to PS administration, two patients were unable to complete the test due to postural symptoms, terminating after 2 and 5 min, respectively; 1 h following PS administration, only one of these patients discontinued the test prematurely. On average patients exhibited a median orthostatic heart rate change of 17 bpm (IQR: 13–23 bpm), calculated as the difference between the maximum heart rate while standing and the heart rate in the supine position. One hour after PS administration the orthostatic heart rate change was significantly lower with 13 bpm (IQR: 9 bpm – 20 bpm, *p* = 0.017) compared to assessment without PS administration, as shown in Figure 2.

### 3.4 Besinger score

The Besinger score demonstrated muscle weakness in all patients, with a median total score of 0.9 points (p) (IQR: 0.7 p − 1.3 p). Among the specific tests, the leg-holding test revealed the most significant weakness, with a median score of 2.0 p (IQR: 2.0 p – 2.5 p). This was followed by the head-holding test, with a median score of 1.5 p (IQR: 1.0 p − 2.0 p), and the arm-holding test, which had a median score of 1.0 p.

### 3.5 MG autoantibodies

None of the patients tested positive for myasthenia gravis specific autoantibodies. No meaningful correlations were identified between mean HGS, PST results, and the questionnaire scores.

## 4 Discussion

This study demonstrates that PS can rapidly improve HGS in patients with ME/CFS. Compared to the decrease in HGS observed upon repeat measurement, HGS increased 1 h after PS administration by approximately 50%. Additionally, PS administration significantly reduced orthostatic heart rate change.

The decline in HGS observed in our study after repeated measurements without PS administration aligns with our previous studies (Jäkel et al., 2021). In these studies also correlations between lower HGS and key clinical symptoms, such as muscle pain, cognitive impairment and disease severity were found. Similarly, Nacul et al., 2018 documented an association between diminished HGS and increased severity of clinical symptoms in ME/CFS, reinforcing the relevance of HGS as an objective marker in this condition (Paffrath et al., 2024).

Remarkably, similar to MG PS administration resulted in rapid increase in muscle strength (Maggi and Mantegazza, 2011). In MG, the mechanism by which PS enhances muscle strength is considered by increasing ACh availability in the neuromuscular junction, counteracting the impaired signal transmission caused by autoantibodies targeting nicotine ACh receptors (Maggi and Mantegazza, 2011). In contrast to MG patients, Glikson et al. demonstrated that PS does not affect muscle strength in healthy individuals, suggesting that this effect is negligible when sufficient ACh is available and the muscle function is intact (Glikson et al., 1991). ME/CFS patients on the other hand have been shown to exhibit structural as well as functional abnormalities in skeletal muscle (Bizjak et al., 2024; Appelman et al., 2024; Wirth and Scheibenbogen, 2021). We found no autoantibodies targeting nicotine ACh receptors in our ME/CFS cohort.

By increasing ACh levels, PS could improve the ability of motor neurons to stimulate muscle fibers and allow for more motor units to be recruited during muscle contractions and, as a result, help improve muscle strength. PS affects neuromuscular transmission but can also modulate parasympathetic activity. Qin et al. demonstrated that PS attenuated the impairment of peripheral and maintained endothelial ultrastructural integrity in in myocardial infarction rat models while also increasing nitric oxide bioavailability and promoting vagal activity (Qin et al., 2014). Given the mechanism of muscle hypoperfusion, due to impaired microcirculation and endothelial dysfunction in ME/CFS (Scheibenbogen and Wirth, 2025), PS might increase blood flow, thus reducing ischemia and improving oxygen and nutrient delivery to the muscles, too. This could result in better muscle performance and reduced fatigue during exertion. Additionally, an RCT by Joseph et al. showed that PS improves perfusion by increasing preload and cardiac output, thereby enhancing muscle perfusion through overall improvements in systemic circulation (Joseph et al., 2022). However, mitochondrial dysfunction in ME/CFS is likely to limit the effectiveness of PS in improving muscle strength, resulting in only a modest effect, as energy production in muscle cells would remain insufficient. Additionally, a study by Baine et al. provides further insight into a possible mechanism of action of PS that may be relevant to ME/CFS. In a mouse model of heart failure, they demonstrated that PS improved cardiac function and rhythmicity by stabilizing ryanodine receptor 2 (RyR2)-mediated calcium release from the sarcoplasmic reticulum and inhibiting STIM1-mediated calcium entry (Baine et al., 2021). These findings support the hypothesis that PS may exert beneficial effects in ME/CFS by modulating intracellular calcium handling, which is known to be disrupted in this condition. Although speculative, altered calcium dynamics may contribute to muscle fatigue and PEM in ME/CFS, and PS might help restore calcium homeostasis and thereby improve muscle performance. This potential mechanism warrants further investigation in future studies.

The reduction in HR increase upon standing observed in our study following PS administration is consistent with findings by Raj et al., 2005, who demonstrated that PS significantly reduced tachycardia and alleviated symptom burden in patients with POTS. Further it is in line with the effect of a single dose of 60 mg PS in ME/CFS which has resulted in improvements in peak exercise oxygen uptake, increased cardiac output and enhanced right ventricular filling pressures (Joseph et al., 2022).

Our study demonstrated moderate but significant short-term effects of PS administration on both HGS and orthostatic heart rate increase. Controlled clinical trials of PS treatment are needed to assess the effects on symptom improvement and clinical outcome in ME/CFS patients as well as to assess potential side effects of PS-treatment such as hypersalivation and gastrointestinal cramping and rarely bronchospasm or coronary spasm. A RCT of PS in ME/CFS patients with orthostatic intolerance is currently being conducted at the Brigham and Women’s Hospital, Harvard Medical School (NCT06366724).

The administration of PS significantly improved muscle strength in the repeated testing after 1 h in our cohort of ME/CFS patients, thus not allowing to differentiate ME/CFS from MG. All ME/CFS patients tested negative for MG-specific autoantibodies, while only 6% to 10% of MG patients have negative results on standard assays for AChR, MuSK, and LRP4 antibodies, referred to as having “seronegative MG.” While patients with seronegative MG are more likely to present with primarily ocular symptoms, they generally share similar features as those with seropositive MG (Deymeer et al., 2007). Additionally, the Besinger score is not a suitable diagnostic tool for differentiating ME/CFS from MG, as both conditions can result in pathological scores due to their overlapping symptoms (Besinger et al., 1983). In a study by Rassler et al. (2011), a mean Besinger score of 0.7 (± 0.1) was reported in patients with mild to moderate MG. In ME/CFS cases with atypical clinical presentation, electrodiagnostic studies are therefore suggested to further rule out the diagnosis of MG (Tankisi et al., 2025).

Limitations of our study include the small sample size and the non-controlled design without randomization of the treatment order and the lack of a control group.

## 5 Conclusion

In conclusion, this study provides evidence that PS can rapidly improve HGS as measure of muscular fatigue in patients with ME/CFS, in contrast to the decrease observed 1 h after repeated measurement. Furthermore, PS significantly reduced the standing heart rate increase in ME/CFS patients, regardless of the presence of POTS. These findings highlight that PS may have the potential to improve key dysregulations in ME/CFS and warrant further investigation in controlled trials.

## Data Availability

The raw data supporting the conclusions of this article will be made available by the authors, without undue reservation.

## References

[ref1] Appelman B. Charlton B. T. Goulding R. P. Kerkhoff T. J. Breedveld E. A. Noort W.. (2024). Muscle abnormalities worsen after post-exertional malaise in long COVID. Nat. Commun. 15:17. doi: 10.1038/s41467-023-44432-3, PMID: 38177128 PMC10766651

[ref2] Baine S. Bonilla I. Belevych A. Stepanov A. Dorn L. E. Terentyeva R.. (2021). Pyridostigmine improves cardiac function and rhythmicity through RyR2 stabilization and inhibition of STIM1-mediated calcium entry in heart failure. J. Cell. Mol. Med. 25, 4637–4648. doi: 10.1111/jcmm.16356, PMID: 33755308 PMC8107086

[ref3] Bateman L. Bested A. C. Bonilla H. F. Chheda B. V. Chu L. Curtin J. M.. (2021). Myalgic encephalomyelitis/chronic fatigue syndrome: essentials of diagnosis and management. Mayo Clin. Proc. 96, 2861–2878. doi: 10.1016/j.mayocp.2021.07.004, PMID: 34454716

[ref4] Bell D. S., The doctor's guide to chronic fatigue syndrome: Understanding, treating, and living with CFIDS. Reading, MA: Da Capo Press Inc; (1995).

[ref5] Berlit P. (2006). Memorix Neurologie, vol. 4. Stuttgart: Georg Thieme Verlag KG.

[ref6] Bertinat R. Villalobos-Labra R. Hofmann L. Blauensteiner J. Sepúlveda N. Westermeier F. (2022). Decreased NO production in endothelial cells exposed to plasma from ME/CFS patients. Vasc. Pharmacol. 143:106953. doi: 10.1016/j.vph.2022.106953, PMID: 35074481

[ref7] Besinger U. A. Toyka K. V. Hömberg M. Heininger K. Hohlfeld R. Fateh-Moghadam A. (1983). Myasthenia gravis: long-term correlation of binding and bungarotoxin blocking antibodies against acetylcholine receptors with changes in disease severity. Neurology 33, 1316–1321. doi: 10.1212/WNL.33.10.1316, PMID: 6684226

[ref8] Bizjak D. A. Ohmayer B. Buhl J. L. Schneider E. M. Walther P. Calzia E.. (2024). Functional and morphological differences of muscle mitochondria in chronic fatigue syndrome and post-COVID syndrome. Int. J. Mol. Sci. 25:1675. doi: 10.3390/ijms25031675, PMID: 38338957 PMC10855807

[ref9] Carruthers B. M. Jain A. K. de Meirleir K. L. Peterson D. L. Klimas N. G. Lerner A. M.. (2003). Myalgic encephalomyelitis/chronic fatigue syndrome. J. Chron. Fatigue Syndr. 11, 7–115. doi: 10.1300/J092v11n01_02

[ref10] Committee on the Diagnostic Criteria for Myalgic Encephalomyelitis/Chronic Fatigue Syndrome; Board on the Health of Select Populations; Institute of Medicine (2015). Beyond Myalgic encephalomyelitis/chronic fatigue syndrome: Redefining an illness. Washington, D. C.: National Academies Press (US). 25695122

[ref11] Cotler J. Holtzman C. Dudun C. Jason L. A. (2018). A brief questionnaire to assess post-exertional malaise. Diagnostics 8:66. doi: 10.3390/diagnostics8030066, PMID: 30208578 PMC6165517

[ref12] Deymeer F. Gungor-Tuncer O. Yılmaz V. Parman Y. Serdaroglu P. Ozdemir C.. (2007). Clinical comparison of anti-MuSK- vs anti-AChR-positive and seronegative myasthenia gravis. Neurology 68, 609–611. doi: 10.1212/01.wnl.0000254620.45529.97, PMID: 17310034

[ref13] Farmakidis C. Pasnoor M. Dimachkie M. M. Barohn R. J. (2018). Treatment of myasthenia gravis. Neurol. Clin. 36, 311–337. doi: 10.1016/j.ncl.2018.01.011, PMID: 29655452 PMC6690491

[ref14] Glikson M. Achiron A. Ram Z. Ayalon A. Karni A. Sarova-Pinchas I.. (1991). The influence of pyridostigmine administration on human neuromuscular functions?Studies in healthy human subjects. Fundam. Appl. Toxicol. 16, 288–298. doi: 10.1016/0272-0590(91)90113-I, PMID: 1647337

[ref15] Hartwig J. Sotzny F. Bauer S. Heidecke H. Riemekasten G. Dragun D.. (2020). IgG stimulated β2 adrenergic receptor activation is attenuated in patients with ME/CFS. Brain. Behav. Immun. Health 3:100047. doi: 10.1016/j.bbih.2020.100047, PMID: 34589837 PMC8474590

[ref16] Jäkel B. Kedor C. Grabowski P. Wittke K. Thiel S. Scherbakov N.. (2021). Hand grip strength and fatigability: correlation with clinical parameters and diagnostic suitability in ME/CFS. J. Transl. Med. 19:159. doi: 10.1186/s12967-021-02774-w, PMID: 33874961 PMC8056497

[ref17] Joseph P. Arevalo C. Oliveira R. K. F. Faria-Urbina M. Felsenstein D. Oaklander A. L.. (2021). Insights from invasive cardiopulmonary exercise testing of patients with Myalgic encephalomyelitis/chronic fatigue syndrome. Chest 160, 642–651. doi: 10.1016/j.chest.2021.01.082, PMID: 33577778 PMC8727854

[ref18] Joseph P. Pari R. Miller S. Warren A. Stovall M. C. Squires J.. (2022). Neurovascular dysregulation and acute exercise intolerance in Myalgic encephalomyelitis/chronic fatigue syndrome: a randomized placebo-controlled trial of pyridostigmine. Chest 162, 1116–1126. doi: 10.1016/j.chest.2022.04.146, PMID: 35526605

[ref19] Kaminski H. J. Kusner L. L. (2018). Myasthenia gravis and related disorders, vol. 3. Cham: Springer International Publishing.

[ref20] Kedor C. Freitag H. Meyer-Arndt L. Wittke K. Hanitsch L. G. Zoller T.. (2022). A prospective observational study of post-COVID-19 chronic fatigue syndrome following the first pandemic wave in Germany and biomarkers associated with symptom severity. Nat. Commun. 13:5104. doi: 10.1038/s41467-022-32507-6, PMID: 36042189 PMC9426365

[ref21] Legler F. Meyer-Arndt L. Mödl L. Kedor C. Freitag H. Stein E.. (2023). Long-term symptom severity and clinical biomarkers in post-COVID-19/chronic fatigue syndrome: results from a prospective observational cohort. EClinicalMedicine 63:102146. doi: 10.1016/j.eclinm.2023.102146, PMID: 37662515 PMC10469383

[ref22] Maggi L. Mantegazza R. (2011). Treatment of myasthenia gravis: focus on pyridostigmine. Clin. Drug Investig. 31, 691–701. doi: 10.2165/11593300-000000000-00000, PMID: 21815707

[ref23] Maksoud R. Magawa C. Eaton-Fitch N. Thapaliya K. Marshall-Gradisnik S. (2023). Biomarkers for myalgic encephalomyelitis/chronic fatigue syndrome (ME/CFS): a systematic review. BMC Med. 21:189. doi: 10.1186/s12916-023-02893-9, PMID: 37226227 PMC10206551

[ref24] Mantegazza R. Cavalcante P. (2019). Diagnosis and treatment of myasthenia gravis. Curr. Opin. Rheumatol. 31, 623–633. doi: 10.1097/BOR.0000000000000647, PMID: 31385879

[ref25] Nacul L. Authier F. J. Scheibenbogen C. Lorusso L. Helland I. B. Martin J. A.. (2021). European network on Myalgic encephalomyelitis/chronic fatigue syndrome (EUROMENE): Expert consensus on the diagnosis, service provision, and Care of People with ME/CFS in Europe. Medicina 57. doi: 10.3390/medicina57050510 PMC8161074 34069603

[ref26] Nacul L. C. Mudie K. Kingdon C. C. Clark T. G. Lacerda E. M. (2018). Hand grip strength as a clinical biomarker for ME/CFS and disease severity. Front. Neurol. 9:992. doi: 10.3389/fneur.2018.00992, PMID: 30538664 PMC6277492

[ref27] Newton D. J. Kennedy G. Chan K. K. F. Lang C. C. Belch J. J. F. Khan F. (2012). Large and small artery endothelial dysfunction in chronic fatigue syndrome. Int. J. Cardiol. 154, 335–336. doi: 10.1016/j.ijcard.2011.10.030, PMID: 22078396

[ref28] Paffrath A. Kim L. Kedor C. Stein E. Rust R. Freitag H.. (2024). Impaired hand grip strength correlates with greater disability and symptom severity in post-COVID Myalgic encephalomyelitis/chronic fatigue syndrome. J. Clin. Med. 13:2153. doi: 10.3390/jcm13072153, PMID: 38610918 PMC11012649

[ref29] Petter E. Scheibenbogen C. Linz P. Stehning C. Wirth K. Kuehne T.. (2022). Muscle sodium content in patients with Myalgic encephalomyelitis/chronic fatigue syndrome. J. Transl. Med. 20:580. doi: 10.1186/s12967-022-03616-z, PMID: 36494667 PMC9733289

[ref30] Qin F. Lu Y. He X. Zhao M. Bi X. Yu X.. (2014). Pyridostigmine prevents peripheral vascular endothelial dysfunction in rats with myocardial infarction. Clin. Exp. Pharmacol. Physiol. 41, 202–209. doi: 10.1111/1440-1681.12198, PMID: 24471445

[ref31] Raj S. R. Black B. K. Biaggioni I. Harris P. A. Robertson D. (2005). Acetylcholinesterase inhibition improves tachycardia in postural tachycardia syndrome. Circulation 111, 2734–2740. doi: 10.1161/CIRCULATIONAHA.104.497594, PMID: 15911704

[ref32] Rassler B. Marx G. Hallebach S. Kalischewski P. Baumann I. (2011). Long-term respiratory muscle endurance training in patients with myasthenia gravis: first results after four months of training. Autoimmune Dis 2011:808607. doi: 10.4061/2011/808607 21869926 PMC3159986

[ref33] Restivo D. A. Centonze D. Alesina A. Marchese-Ragona R. (2020). Myasthenia gravis associated with SARS-CoV-2 infection. Ann. Intern. Med. 173, 1027–1028. doi: 10.7326/L20-0845, PMID: 32776781 PMC7429993

[ref34] Scheibenbogen C. Wirth K. J. (2025). Key pathophysiological role of skeletal muscle disturbance in post COVID and Myalgic encephalomyelitis/chronic fatigue syndrome (ME/CFS): accumulated evidence. J. Cachexia Sarcopenia Muscle 16:e13669. doi: 10.1002/jcsm.13669, PMID: 39727052 PMC11671797

[ref35] Shah S. M. I. Yasmin F. Memon R. S. Jatoi N. N. Savul I. S. Kazmi S.. (2022). COVID-19 and myasthenia gravis: a review of neurological implications of the SARS-COV-2. Brain Behav. 12:e2789. doi: 10.1002/brb3.2789, PMID: 36306401 PMC9759145

[ref36] Sheldon R. S. Grubb B. P. II Olshansky B. Shen W. K. Calkins H. Brignole M.. (2015). 2015 heart rhythm society expert consensus statement on the diagnosis and treatment of postural tachycardia syndrome, inappropriate sinus tachycardia, and vasovagal syncope. Heart Rhythm 12, e41–e63. doi: 10.1016/j.hrthm.2015.03.029, PMID: 25980576 PMC5267948

[ref37] Słomko J. Estévez-López F. Kujawski S. Zawadka-Kunikowska M. Tafil-Klawe M. Klawe J. J.. (2020). Autonomic phenotypes in chronic fatigue syndrome (CFS) are associated with illness severity: a cluster analysis. J. Clin. Med. 9:2531. doi: 10.3390/jcm9082531, PMID: 32764516 PMC7464864

[ref38] Sotzny F. Blanco J. Capelli E. Castro-Marrero J. Steiner S. Murovska M.. (2018). Myalgic encephalomyelitis/chronic fatigue syndrome-evidence for an autoimmune disease. Autoimmun. Rev. 17, 601–609. doi: 10.1016/j.autrev.2018.01.009, PMID: 29635081

[ref39] Spence V. A. Khan F. Kennedy G. Abbot N. C. Belch J. J. F. (2004). Acetylcholine mediated vasodilatation in the microcirculation of patients with chronic fatigue syndrome. Prostaglandins Leukot. Essent. Fatty Acids 70, 403–407. doi: 10.1016/j.plefa.2003.12.016, PMID: 15041034

[ref40] Taheri A. Davoodi L. Soleymani E. Ahmadi N. (2022). New-onset myasthenia gravis after novel coronavirus 2019 infection. Respirol Case Rep 10:e0978. doi: 10.1002/rcr2.978, PMID: 35620352 PMC9125167

[ref41] Tankisi H. Pugdahl K. Johnsen B. Camdessanché J. P. de Carvalho M. Fawcett P.. (2025). Electrodiagnostic criteria for neuromuscular transmission disorders suggested by a European consensus group. Clin. Neurophysiol. Pract. 10, 79–83. doi: 10.1016/j.cnp.2025.02.011, PMID: 40129481 PMC11931287

[ref42] van Campen C. L. M. C. Rowe P. C. Visser F. C. (2018). Low sensitivity of abbreviated tilt table testing for diagnosing postural tachycardia syndrome in adults with ME/CFS. Front. Pediatr. 6:349. doi: 10.3389/fped.2018.00349, PMID: 30505831 PMC6250822

[ref43] van Campen C. L. M. C. Verheugt F. W. A. Rowe P. C. Visser F. C. (2020). Cerebral blood flow is reduced in ME/CFS during head-up tilt testing even in the absence of hypotension or tachycardia: a quantitative, controlled study using Doppler echography. Clin. Neurophysiol. Pract. 5, 50–58. doi: 10.1016/j.cnp.2020.01.003, PMID: 32140630 PMC7044650

[ref44] Vernino S. Bourne K. M. Stiles L. E. Grubb B. P. Fedorowski A. Stewart J. M.. (2021). Postural orthostatic tachycardia syndrome (POTS): state of the science and clinical care from a 2019 National Institutes of Health expert consensus meeting-part 1. Auton. Neurosci. 235:102828. doi: 10.1016/j.autneu.2021.102828, PMID: 34144933 PMC8455420

[ref45] Ware J. E. (2000). SF-36 health survey update. Spine (Phila Pa 1976) 25, 3130–3139. doi: 10.1097/00007632-200012150-00008, PMID: 11124729

[ref46] Wirth K. J. Scheibenbogen C. (2021). Pathophysiology of skeletal muscle disturbances in Myalgic encephalomyelitis/chronic fatigue syndrome (ME/CFS). J. Transl. Med. 19:162. doi: 10.1186/s12967-021-02833-2, PMID: 33882940 PMC8058748

