# Supplementary material for: Pyridostigmine improves hand grip strength in patients with myalgic encephalomyelitis/chronic fatigue syndrome
Source: Front Neurosci. 2025 Sep 3;19:1637838. doi: 10.3389/fnins.2025.1637838 (PMC12441162; doi:10.3389/fnins.2025.1637838)
Supplement: Supplementary file 1 [file Table_1.DOCX]

Supplementary Material

# Supplementary Figures and Tables

## Supplementary Tables

Table S1: cutoff values for decreased hand grip strength according to the digital hand dynamometer`s manufacturer (EH101, Deyard, Shenzhen, China)

| Agegroup (years) | normal range of hand grip strength in kg | | | | | |
| --- | --- | --- | --- | --- | --- | --- |
|  | Male | | | | Female | |
| 18-19 |  | | 35.7-55.5 |  | | 19.2-31.0 |
| 20-24 |  | | 36.8-56.6 |  | | 21.5-35.3 |
| 25-29 |  | | 37.7-57.5 |  | | 25.6-41.4 |
| 30-34 |  | | 36.0-55.8 |  | | 21.5-35.3 |
| 35-39 |  | | 35.8-55.6 |  | | 20.3-34.1 |
| 40-44 |  | | 35.5-55.3 |  | | 18.9-32.7 |
| 45-49 |  | 34.7-54.5 | |  | | 18.6-32.4 |
| 50-54 |  | 32.9-50.7 | |  | | 18.1-31.9 |
| 55-59 |  | 30.7-48.5 | |  | | 17.7-31.5 |
| 60-64 |  | 30.2-48.0 | |  | | 17.2-31.0 |
| 65-69 |  | 28.2-44.0 | | |  | 15.4-27.2 |
